# Prospectively defined murine mesenchymal stem cells inhibit *Klebsiella pneumoniae*-induced acute lung injury and improve pneumonia survival

**DOI:** 10.1186/s12931-015-0288-1

**Published:** 2015-10-06

**Authors:** Holger Hackstein, Anne Lippitsch, Philipp Krug, Inna Schevtschenko, Sabine Kranz, Matthias Hecker, Kristina Dietert, Achim D. Gruber, Gregor Bein, Cornelia Brendel, Nelli Baal

**Affiliations:** Institute for Clinical Immunology and Transfusion Medicine, Universities of Giessen and Marburg Lung Center (UGMLC), Member of the German Center for Lung Research (DZL), University Hospital Giessen und Marburg, Justus-Liebig-University Giessen, Langhansstr. 7, D-35390 Giessen, Germany; Department of Internal Medicine II, Universities of Giessen and Marburg Lung Center (UGMLC), Member of the German Center for Lung Research (DZL), University Hospital Giessen und Marburg, Justus-Liebig-University Giessen, Giessen, Germany; Department of Veterinary Pathology, Freie Universität Berlin, Robert-von-Ostertag-Str. 15, 14163, Berlin, Germany; Department of Hematology, Oncology and Immunology, Philipps University Marburg, University Hospital Giessen und Marburg, Marburg, Germany

**Keywords:** Pneumonia, Mesenchymal stem cells, Klebsiella pneumonia, Acute lung injury

## Abstract

**Background:**

Numerous studies have described the immunosuppressive capacity of mesenchymal stem cells (MSC) but these studies use mixtures of heterogeneous progenitor cells for in vitro expansion. Recently, multipotent MSC have been prospectively identified in murine bone marrow (BM) on the basis of PDFGRa^+^ SCA1^+^ CD45^−^ TER119^−^ (PαS) expression but the immunomodulatory capacity of these MSC is unknown.

**Methods:**

We isolated PαS MSC by high-purity FACS sorting of murine BM and after in vitro expansion we analyzed the in vivo immunomodulatory activity during acute pneumonia. PαS MSC (1 × 10^6^) were applied intratracheally 4 h after acute respiratory *Klebsiella pneumoniae* induced infection.

**Results:**

PαS MSC treatment resulted in significantly reduced alveolitis and protein leakage in comparison to mock-treated controls. PαS MSC-treated mice exhibited significantly reduced alveolar TNF-α and IL-12p70 expression, while IL-10 expression was unaffected. Dissection of respiratory dendritic cell (DC) subsets by multiparameter flow cytometry revealed significantly reduced lung DC infiltration and significantly reduced CD86 costimulatory expression on lung CD103^+^ DC in PαS MSC-treated mice. In the post-acute phase of pneumonia, PαS MSC-treated animals exhibited significantly reduced respiratory IL-17^+^ CD4^+^ T cells and IFN-γ^+^ CD4^+^ T cells. Moreover, PαS MSC treatment significantly improved overall pneumonia survival and did not increase bacterial load.

**Conclusion:**

In this study we demonstrated for the first time the feasibility and in vivo immunomodulatory capacity of prospectively defined MSC in pneumonia.

**Electronic supplementary material:**

The online version of this article (doi:10.1186/s12931-015-0288-1) contains supplementary material, which is available to authorized users.

## Background

The use of surface markers, such as CD34 for the prospective identification and isolation of hematopoietic stem cells (HSC) has fundamentally improved the clinical development and standardization of stem cell transplantation [1–3]. In contrast, most, if not all knowledge regarding the immunosuppressive capacity of mesenchymal stem cells (MSC) derives from studies using starting cultures of mixed cell populations [4, 5]. Therefore, the International Society for Cellular Therapy has published criteria for the definition of in vitro expanded heterogeneous MSC including positivity for CD73, CD90 and CD105, negativity for hematopoietic surfaces markers and other parameters [6]. Although these criteria are helpful to facilitate the comparison of heterogeneous MSC cultures, the prospective identification and direct isolation of homogenous MSC populations on the basis of surface markers will improve the standardization, comparability and reproducibility of MSC research.

Morikawa et al. has recently identified surface markers for the prospective identification of purified murine multipotent MSC from adult BM [7, 8]. Their results suggested that the co-expression of Platelet-derived growth factor receptor α (PDFGRα, CD140a) and stem-cell antigen 1 (Sca-1) on CD45 and TER119 negative BM cells effectively identifies primary MSC and these were termed double positive PDFGRa^+^ SCA1^+^ CD45^−^ TER119^−^ (PαS) MSC [7]. PαS MSC exhibited the highest numbers of colonies in a colony-forming unit-fibroblasts (CFU-F) assay and represented the only BM subset yielding MSC typical fibroblast/spindle-shaped cells. Only PαS cultures demonstrated a potent multilineage differentiation capacity into adipogenic, chondrogenic and osteogenic lineages indicating a high enrichment for MSC [7].

Heterogeneous results have been reported with regard to the immunosuppressive capacity of MSC [9, 10]. With respect to T cells, different pathways have been suggested including the direct inhibition of T effector cell proliferation and promotion of regulatory T cells [5]. Similarly, MSC have been suggested to inhibit the cytotoxicity of NK cells and CD8^+^ T lymphocytes [11, 12]. With respect to dendritic cells (DC), MSC inhibit the monocyte-derived DC differentiation and suppress the costimulatory molecule expression, the pro-inflammatory cytokines IL12p70 and TNF-α as well as the priming of responder T cells [5, 13, 14].

However, an increasing number of reports indicate that MSC are not immunosuppressive by themselves but can also exert immunostimulatory activity. For instance, Waterman et al. suggested that MSC can be polarized in immunosuppressive MSC1 or pro-inflammatory MSC2 by TLR3 and TLR4 triggering [15]. Moreover, some groups have reported accelerated heart allograft rejection and a failure to inhibit graft-versus-host disease after MSC treatment [16, 17]. Potential mechanisms of immunostimulatory MSC may be related to the release of activatory exosomes or CCL2 production.

With respect to these heterogeneous immunological results and the increasing number of MSC studies suggesting therapeutic activity in acute pneumonia [18–20] the objective of this study was to investigate the in vivo immunomodulatory capacity of prospectively defined MSC in an animal model of gram negative pneumonia. Furthermore, to address more precisely the impact of this treatment on respiratory cellular inflammation we have used multiparameter flow cytometry allowing the dissection of key respiratory leukocyte subsets after MSC therapy [21–23].

## Methods

### Mice, *Klebsiella pneumoniae* infection and MSC treatment

Specific-pathogen-free C57BL/6 (C57BL/6NCrl) and B6.Cg-Tg(TcraTcrb)425Cbn/J mice (20–25 g each) were purchased from Charles River, Germany and maintained under specific-pathogen-free conditions. The mice were infected intratracheally with 3.5 × 10^5^ CFU of *Klebsiella pneumoniae* (*K. pneumoniae*) serotype 2 (*American Type Culture Collection* (ATCC) 43816) in 50 μl sterile 0.9 % NaCl as previously described [23]. Four hours p.i. the anesthetized mice received 1 × 10^6^ washed PαS MSC in 50 μl 0.9 % NaCl intratracheally and were analyzed at the indicated time points (KpN/MSC). The control mice were treated identically but received 50 μl 0.9 % NaCl (KpN/NaCl). Some mice received 1 × 10^6^ washed mouse lung fibroblasts (MLg;ATCC CCL-206) in 50 μl 0.9 % NaCl and were indicated as such (KpN/MLg). Experiments were approved by the regional animal authority board (#75/2011).

### BM preparation, PαS MSC sorting and in vitro expansion

Femura and tibiae were prepared as previously described with minor modifications [7]. The bone fragments were collected and digested for 1 h at 37 °C in alpha-MEM with L-Glutamine (PAN Biotech, Germany), 10 % FBS (PAA, Germany), 1 % penicillin/streptomycin (PAN Biotech) containing 3.92 U/ml collagenase (Wako Chemicals, Japan), 10 mM Hepes (Gibco, Germany) and 3 mM CaCl2. The cell suspension was filtered through a 70 μm cell strainer (BD Falcon, Germany) and collected by centrifugation at 400 g for 5 min at 4 °C. Red blood cells were lysed using 155 mM NH_4_Cl/10 mM KHCO_3_ buffer (pH 7.4) and washed with HBSS (PAN Biotech, Germany). After digestion, leucocytes were depleted with CD45 magnetic beads (Miltenyi Biotech, Germany) and stained with fluorochrome-labelled monoclonal antibodies and sorted by a BD ARIAIII cell sorter (Becton Dickinson, San Jose, CA, USA). PαS MSC were defined as positive for CD140a and Sca-1 and negative for CD45 and TER119 and were expanded in PureCoat Amine plates/flasks (BD, Germany) in alpha-MEM medium supplemented with L-Glutamine, 5 % FBS (mesenchymal stem cell-qualified, Life technologies, Germany), and 5 % human platelet lysate. Medium was changed every 3–7 days depending on cell growth. Human platelet lysate was prepared as previously described [24].

### Lung preparation

Lung single cell suspensions (lung homogenates) were prepared after enzymatic digestion as previously described in detail [23]. In brief, the mice were euthanized and the lungs were perfused via the right ventricle with HBSS (PAA, Germany) to remove the intravascular pool of cells. The tissues were minced and digestion was performed in 0.09 U/ml type A collagenase (Roche, Germany) and 9.09 U/ml DNase (Roche, Germany) in IMDM (PAA, Germany) with 10 % FCS (PAA, Germany) at 37 °C for 1 h. The single cell suspensions were prepared by tissue resuspension with 20 G 1 ½ cannulas (0.9 × 40 mm; BD, Germany) and by mashing through a 70 μM cell strainer (BD, Germany). Red blood cells were lysed by ammonium chloride lysis. The cells were washed with HBSS for flow cytometry staining, or the leukocytes were magnetic-bead sorted after washing with PBS/2 % BSA/2 mM EDTA (PAA, Germany). Bronchoalveolar lavages (BAL) were performed as previously described [25]. The bacterial load in lung homogenates and BAL were defined by preparing a two-fold dilution series in sterile HBSS after centrifugation (2500 g, 15 min, 4 ° C) according to the method developed by Schott [26].

### Flow cytometry

Cellular phenotyping and sorting were performed on a BD ARIAIII cell sorter (Becton Dickinson, San Jose, CA, USA). The following fluorochrome-labelled mAbs conjugated to FITC, PE, PeCy7, PerCPCy5.5, APC, APC-Cy7, Brilliant Violet 510, Brilliant Violet 605, Pacific Blue and Alexa700 or appropriate isotype controls were used for cell surface staining: CD11b, CD11c, CD45 (clone 30-F1), CD86, CD103, CD140a (clone APA5), CD274 (PD-L1), MHC-class II (I-A^b^), GR-1, F4/80, NK1.1, SCA-1 (clone D7), Siglec-F (BD Biosciences, Germany), TER119 and 120G8 (Dendritics, France). The surface staining time was 30 min on ice and cells were washed with staining buffer (1× HBSS, PAA, Germany) at 400 g for 5 min at room temperature (RT) before analysis. The number of acquired events was ≥ 500,000.

The viability of sorted cells was >90 % as indicated by Sytox blue (Life Technologies, Germany) staining. All mAbs were ordered from Biolegend, Germany unless indicated otherwise. Absolute cell counts were determined with AccuCount Fluorescent Particles 7.7 μm (Spherotech, Lake Forest, USA).

### CD4 T cell proliferation assay

CD4+ T cells were isolated from the spleens of OT-II mice (B6.Cg-Tg(TcraTcrb)425Cbn/J) using the CD4+ T Cell Isolation Kit (Miltenyi Biotec, Germany). CD4+ T cells were labeled with 0,5 μM CFSE (eBioscience, Germany) for 15 min at 37 °C with frequent agitation, then washed before being used in proliferation assays. DC were generated from C57BL/6 BM hematopoietic stem cells by culturing for 7 days in 10 ng/ml murine GM-CSF and 10 ng/ml murine IL-4 as previously described [27]. CD4+ T-cells (1 × 10^5^) were cultured with DC (1 × 10^4^) and MSC (1 × 10^4^) in RPMI 1640 containing 10 % FCS (PAA, Germany) for 5 days. Ovalbumin (OVA, 100 μg/ml) protein (Hyglos, Germany) was added to the culture medium. OVA-specific proliferation was evaluated as a CFSE dilution by flow cytometry.

### Intracellular cytokine and Foxp3 staining

For cytokine staining cells were stimulated for 6 h at 37 °C with 50 ng/ml PMA (Sigma-Aldrich, Germany), 1 μg/ml Ionnomycin (Sigma-Aldrich, Germany) and 3 μg/ml brefeldin A (eBioscience, Germany) in RPMI with 10 % FBS (PAA, Germany). For intracellular cytokine or Foxp3 staining samples were first stained for surface antigens, washed with PBS (PAN Biotech, Germany), and centrifuged for 5 min at RT and 400 g. The cell pellets were vortexed for dissociation and incubated with fixation/permeabilization buffer (BD, Germany) 20 min at RT. After washing twice with 2 ml of permeabilization/washing buffer (BD, Germany) the cells were resuspended in 100 μl permeabilization buffer. Intracellular mAbs (IFN-γ, IL-4, IL-10, IL-17 from Biolegend, Germany; Foxp3, eBioscience, Germany) or isotype controls were added at the recommended concentrations and incubated 30 min at RT. Cells were washed two times with permeabilization/washing buffer and immediately analyzed by flow cytometry. The number of acquired events was ≥ 500,000.

### Respiratory leukocyte subset discrimination

The gating strategy has been described recently with minor modifications [23]. Briefly, out of the CD45^+^ cells, neutrophils were identified by GR1^bright^CD11b^bright^ expression. Subsequently, out of the neutrophil negative fraction, macrophages were identified as SiglecF^++^/CD11c positive cells. DC were identified according to CD11c^+^Siglec-F^neg^ NK1.1^neg^ expression to exclude autofluorescent macrophages and NK cells. Then they were further dissected into plasmacytoid DC (120 g8^+^ CD11b^neg^) and after MHC-class II^+^ gating into CD103 DC (CD103^+^ CD11b^neg^) and CD11b DC (CD11b^+^ CD103^neg^). CD3^+^ T cells and CD19^+^ B cells were identified within the CD45^+^ SSC^low^ fraction. Out of the CD3^+^ T cell fraction, CD4^+^ and CD8^+^ T cells were identified on the basis of CD4 and CD8 expression, respectively. NK cells were identified as CD3^neg^ NK1.1^+^ cells and γδ T cells were identified as CD3^+^ γδ TCR^+^ cells (Additional file 1: Figure S1). T regulatory cells were identified as CD3^+^CD4^+^CD25^+^Foxp3^+^ cells.

### BAL protein and cytokine quantification

Protein quantification was performed with Pierce BCA Protein Assay Kit (Thermo Scientific, Germany). Mouse TNF-α, IL-10 and IL-12p70 were quantified by cytometric bead arrays according to the manufacturer’s instructions (Flowcytomix, eBioscience, Germany.

### Statistical analyses

Statistical analyses were performed using the GraphPad Prism software version 5.02 (Graphpad Software, Inc., USA). The significance of any differences between groups were analyzed by the one-way ANOVA and Tukey post-test for multiple comparisons. Survival curve comparison and analysis was performed using the logrank test. A *p*-value of < 0.05 was considered statistically significant.

## Results

### Prospective high-purity PαS MSC isolation and in vitro expansion

**Fig. 1:**
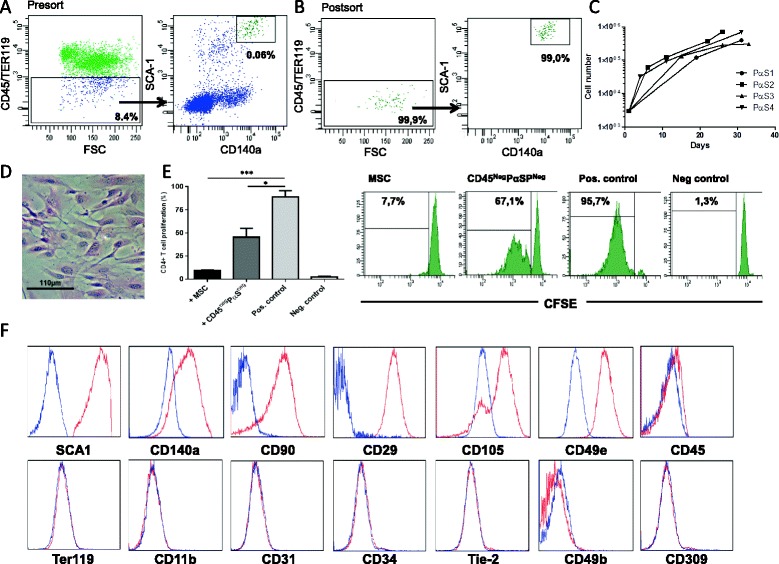
Purification and expansion of PαS MSC. Representative flow cytometry results of PαS MSC before (**a**) and after (**b**) cell sorting. Representative expansion kinetic of 3000 PαS MSC (*n* = 4) (**c**) and phase contrast micrographs of CFU-Fs 28 days after plating (**d**). T-cell inhibitory capacity of PαS MSC versus non-MSC control (“CD45^neg^ PαS^neg^”: CD45^neg^ TER119^neg^ SCA-1^neg^ CD140a^neg^) added to ovalbumin-pulsed DC in a CFSE T cell proliferation assay with OVA-TCR specific CD4+ T cells as described in the Methods section. Positive control are Ovalbumin-pulsed DC plus OVA-TCR specific CD4+ T cells and negative control are DC plus OVA-TCR specific CD4+ T cells without OVA (*n* = 3; (**e**). Representative phenotype of PαS MSC after in vitro expansion before intratracheal application after staining with isotype control (*blue*) and indicated mAbs (*red*; **f**). *p < 0.05, ***p < 0.001; data from *n* ≥ 4 (**a**-**d**), or *n* = 2 (**e**, **f**) experiments

In the BM of C57BL/6 mice less than 0.1 % of nucleated cells exhibited the phenotype of PαS MSC (CD45^neg^ TER119^neg^ CD140a^+^ SCA-1^+^; Fig. 1a). PαS MSC were purified via FACS sorting to a final purity of >98 % (Fig. 1b). Due to the high purity sorting procedure, the cell yield per mouse was low (range 180–400 PαS MSC per mouse). PαS MSC were then grown in vitro for 20–40 days (Fig. 1c). They exhibited a spindle-shaped morphology (Fig. 1d) and inhibited T-cell proliferation in an antigen-specific T proliferation assay with OVA-pulsed DC and transgenic OVA-TCR specific CD4+ responder T cells (Fig. 1e). PαS MSC retained their expression of CD140a (PDFGRα) and SCA-1 (Fig. 1f). In addition, extended phenotypic analysis of expanded PαS MSC revealed homogenous expression of the MSC markers CD29, CD49e, CD90 and CD105 and the absence of the HSC/endothelial marker CD34, the leukocyte markers CD45, CD49b, the endothelial markers CD309, Tie-2 and the erythroid marker TER119 (Fig. 1f).

### PαS MSC inhibit acute alveolitis and lung injury after *K. pneumoniae* infection

**Fig. 2:**
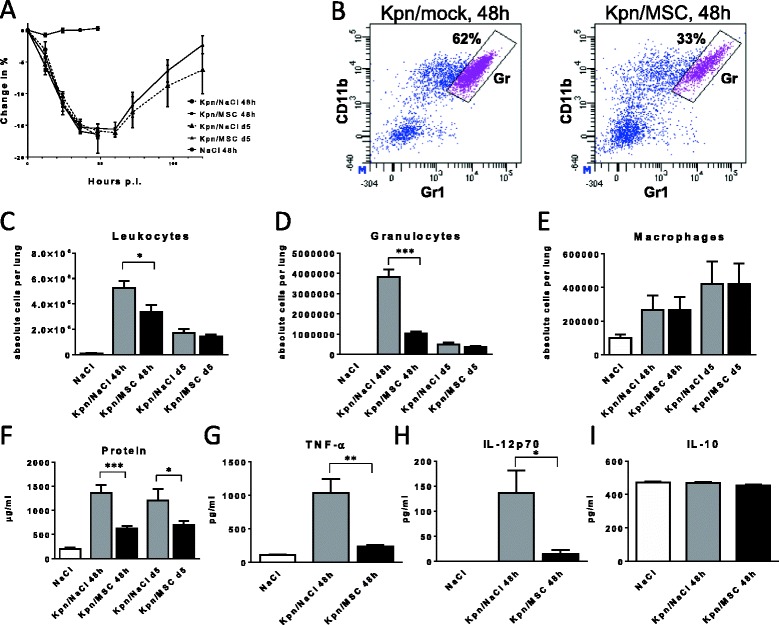
PαS MSC inhibit alveolar TNF-α, IL-12p70 and acute lung injury after *K. pneumoniae* infection. *K. pneumoniae* infected animals were treated with PαS MSC (4 h p.i.). Analysis of body weight (**a**) and flow cytometry-based quantitation of alveolar leukocytes, granulocytes and macrophages (**b**-**e**). Alveolar protein (**f**) and alveolar cytokines TNF-α (**g**), IL-12p70 (**h**) and IL-10 (**i**) were quantified by cytometric bead array. Mean ± SEM; *n* ≥ 3 (**a**, **c**-**e**); *n* ≥ 4 (**f**); *n* ≥ 6 (**g**-**i**). *p < 0.05; **p < 0.01; ***p < 0.001 versus mock-treated infected controls. Data from *n* ≥ 2 (**a**-**i**) experiments. Kpn, infected with *K*. pneumoniae

**Fig. 3:**
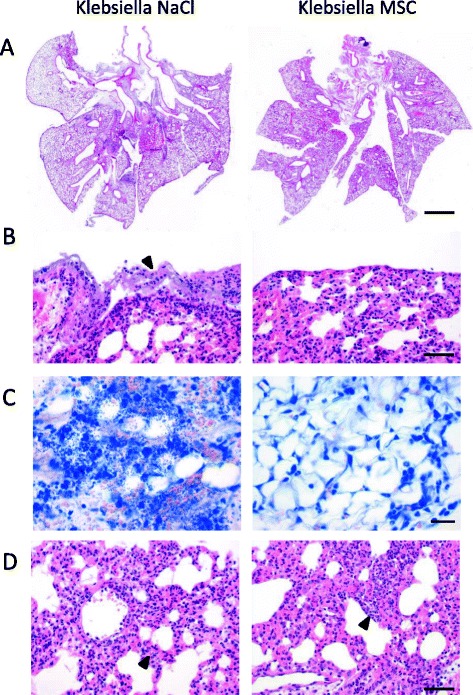
Histopathological analysis of MSC–treated animals after K. pneumoniae infection. Lungs were harvested, fixed in formalin, and embedded in paraffin and 2 μm sections were prepared and stained with hematoxylin and eosin (**a**, **b**, **d**) or Giemsa (**c**) for histopathological analyses of MSC-treated versus mock-treated (NaCl) controls animals after K. pneumoniae infection (d2 p.i.). **a** K. pneumoniae induced suppurative and necrotizing pneumonia with a similar distribution of lung lesions in both groups. **b**, **c** Infected NaCl-treated controls showed severe suppurative and necrotizing pleuritis (**b**, *arrowhead*) and steatitis (**c**), admixed with myriads of bacteria, whilst MSC-treated animals displayed neither pleuritic, steatitis nor bacteria on the lung surface or in the mediastinal fat tissue. **d** In *K. pneumoniae-*infected NaCl-treated controls, neutrophils and histiocytes were predominantly located at the interstitium of the lungs (*arrowhead*) whereas in MCS-treated lungs, neutrophils were also present in alveolar spaces (*arrowhead*) consistent with suppurative bronchopneumonia. Representative images are shown (*n* = 3 experiments). *Bar* (**a**), 2 mm. *Bar* (**b**, **d**), 50 μm. *Bar* (**c**), 20 μm

At 4 h after induction of respiratory *K. pneumoniae* infection, the animals intratracheally received 1 × 10^6^ PαS MSC that were resuspended in NaCl or a negative control (NaCl). Irrespective of PαS MSC treatment, the infected animals exhibited a marked reduction of body weight within 48 h p.i. representing an indirect sign of disease (Fig. 2a). Dissection of major alveolar leukocyte subsets indicated a significant inhibition of acute alveolitis by PαS MSC treatment (Fig. 2b-e). Furthermore, analysis of alveolar protein content as a measure of alveolar protein permeability and fluid clearance revealed that it was significantly reduced indicating less severe acute lung injury (*p* < 0.001, 48 h p.i.; Fig. 2f). Because the protein content represents a rather crude measurement of alveolar injury and inflammation we specifically determined the major pro-inflammatory cytokines TNFα and IL-12p70 and the immunoregulatory cytokine IL-10 (Fig. 2g-i). PαS MSC treatment significantly inhibited both alveolar TNFα (*p* < 0.01) and IL-12p70 (*p* < 0.05) whereas IL-10 was unaffected. These data suggest that PαS MSC treatment inhibited *K. pneumoniae*-induced alveolar cellular inflammation, functional deterioration and pro-inflammatory cytokine production. In addition, histopathological analysis (Fig. 3) indicated severe suppurative and necrotizing pleuritis and steatitis admixed with myriads of bacteria in contrast to MSC-treated animals that did not display pleuritic, steatitis or bacteria on the lung surface or in the mediastinal fat tissue. Bacteriae were almost absent in MSC-treated animals but abundant in the mock-treated controls.

### PαS MSC inhibit respiratory leukocytosis and DC infiltration after *K. pneumoniae* infection

**Fig. 4:**
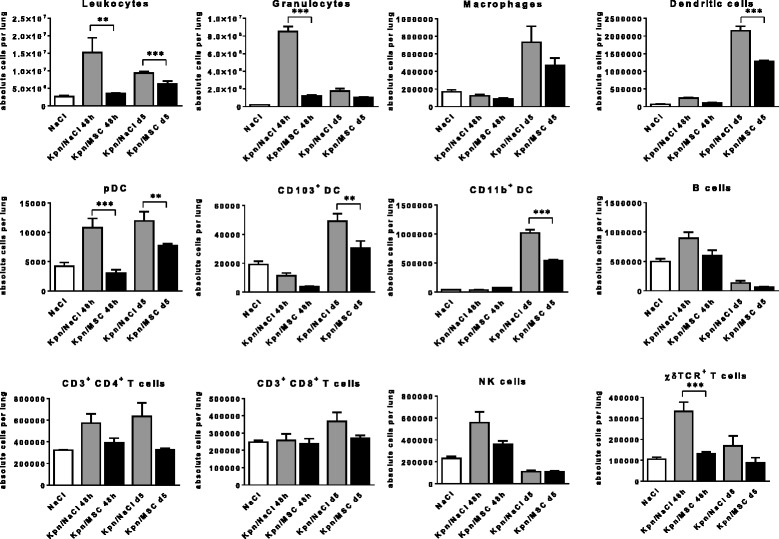
PαS MSC treatment modulates respiratory leukocyte subsets after *K. pneumoniae* infection. Representative flow cytometry analysis of respiratory leukocytes, granulocytes, macrophages, DC, pDC, CD103^+^ DC, CD11b^+^ DC, B cells, CD3^+^ CD4^+^ T cells, CD3^+^ CD8^+^ T cells, NK cells and γδ T cells in lung homogenates of *K. pneumoniae*-infected (Kpn) animals at different timepoints p.i. treated with PαS MSC versus mock-treated infected animals or uninfected.controls. Mean ± SEM; *n* ≥ 6 (48 h p.i.); *n* ≥ 4 (d5 p.i.). **p < 0.01; ***p < 0.001 versus mock-treated infected controls. Data from *n* ≥ 3 experiments

In addition to the isolated alveolar compartment analysis we extended the analysis to all major leukocyte subsets in lung homogenates by using multiparameter flow cytometry during the acute (48 h p.i) and post-acute (d5 p.i) *K. pneumoniae* infection to better understand the modulation of the lung inflammatory process (Fig. 4). PαS MSC treatment significantly suppressed granulocyte (*p* < 0.001, 48 h p.i) and DC infiltration indicating reduced inflammation (*p* < 0.001, d5 p.i). Dissection of respiratory DC subsets into plasmacytoid DC, CD103^+^ DC and CD11b^+^ DC revealed a significant suppression of plasmacytoid DC infiltration early after infection (*p* < 0.001, 48 h p.i) and of plasmacytoid DC, CD103^+^ DC and CD11b^+^ DC late after infection (*p* < 0.01, d5 p.i). In contrast, respiratory B lymphocytes and CD4^+^ and CD8^+^ T lymphocytes were not significantly affected. In addition, with respect to innate lymphocytes, PαS MSC treatment significantly suppressed respiratory γδ-TCR^+^ T cell numbers 48 h p.i. (*p* < 0.001) but not NK cells.

### PαS MSC inhibit CD86 costimulatory molecule expression on lung CD103^+^ DC during acute *K. pneumoniae* infection

**Fig. 5:**
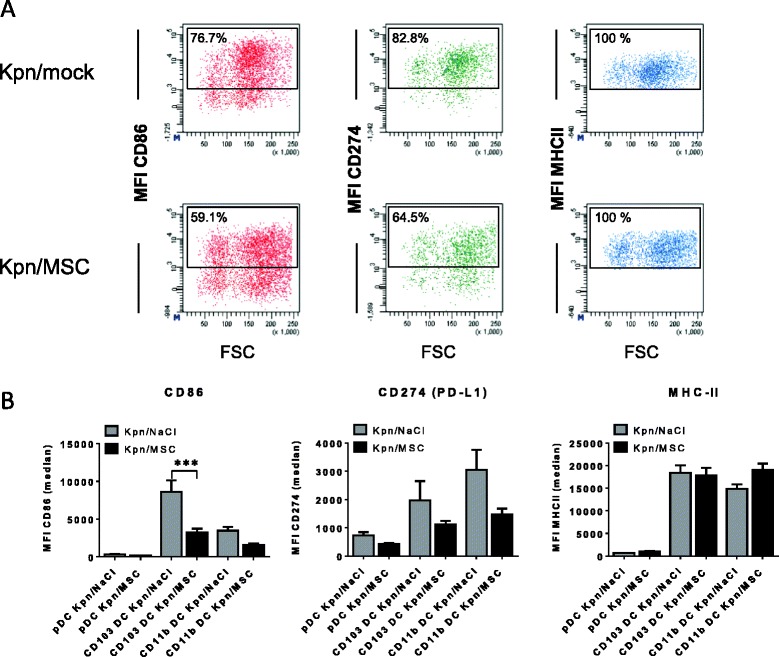
PαS MSC treatment modulates CD86 costimulatory molecule expression on CD103^+^ respiratory DC after *K. pneumoniae* infection. Representative flow cytometry analysis of CD86, CD274 (PD-L1) and MHC-class II expression on CD103^+^ respiratory DC in *K. pneumoniae*-infected animals treated with PαS MSC versus mock-treated infected animals at 48 h p.i. (**a**). Quantitative analysis of median fluorescence intensity (MFI) on respiratory DC subsets in *K. pneumoniae*-infected (Kpn) animals treated with PαS MSC versus controls at 48 h p.i. (**b**). Mean ± SEM; *n* ≥ 6 (48 h p.i.); *n* ≥ 4 (d5 p.i.).***p < 0.001 versus mock-treated infected controls. Data from *n* = 2 (A-B) experiments

Given the observation, that PαS MSC treatment significantly inhibited lung DC infiltration we questioned whether surface expression of the functionally relevant costimulatory molecule CD86, the immunoregulatory molecule PD-L1 (CD274) and MHC-class II were affected by PαS MSC treatment of lung DC subsets (Fig. 5a-b). These analyses revealed a significant suppression of CD86 expression on CD103^+^ DC early after infection (*p* < 0.001, 48 h p.i.) in contrast to later time points (d5 p.i.)

### PαS MSC inhibit post-infectious expansion of lung pro-inflammatory IFN-γ^+^ and IL-17^+^ T cells without affecting respiratory T regulatory cells

**Fig. 6:**
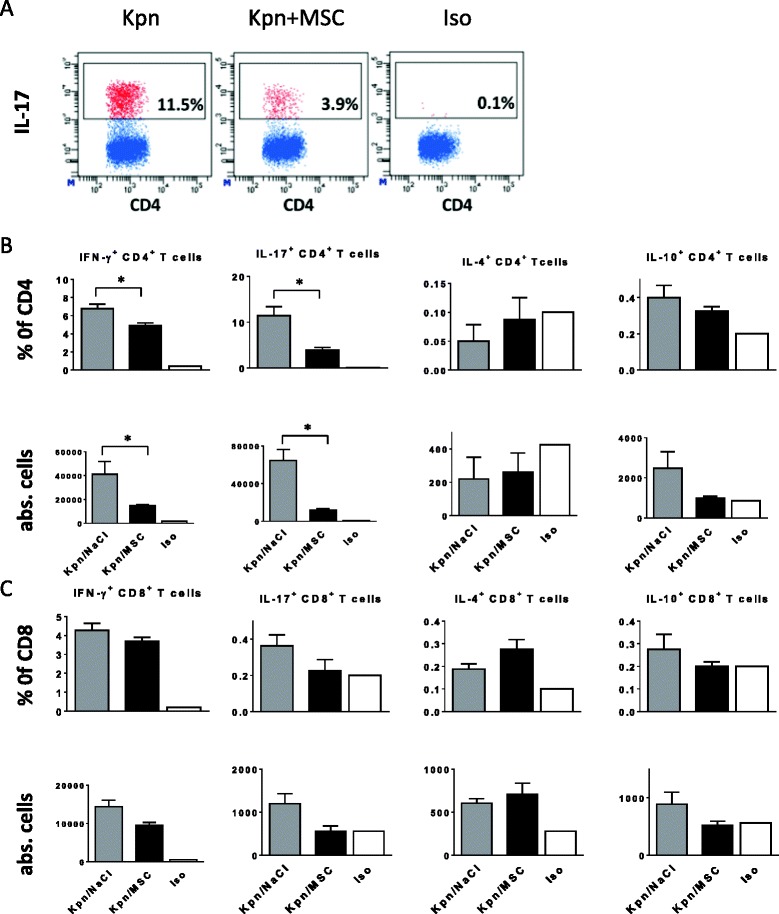
PαS MSC suppress in vivo expansion of respiratory IFN-γ^+^ and IL-17^+^ CD4^+^ T cells after *K. pneumoniae* infection. Representative flow cytometry analysis of intracellular IL-17 expression in respiratory CD4^+^ T cells in *K. pneumoniae*-infected animals treated with PαS MSC versus mock-treated infected animals at d5 p.i. (**a**). Iso refers to isotype-matched fluorescence minus-one controls to assess background staining. Relative frequencies and absolute numbers of cytokine expressing respiratory CD4^+^ and CD8^+^ T cell subsets in *K. pneumoniae*-infected (Kpn) animals treated with PαS MSC versus mock-treated infected animals (**b**-**c**). Mean ± SEM; *n* ≥ 4; *p < 0.05 versus mock-treated infected controls. Data from *n* ≥ 2 (A-C) experiments

The identification of reduced DC infiltration and impaired CD103^+^ DC costimulatory molecule expression raised the question whether PαS MSC treatment interfered also with the post-infectious expansion of polarized T cells (Fig. 6a-c). The results indicated that PαS MSC treatment significantly inhibited both the relative and absolute numbers of lung CD4^+^ IFN-γ^+^ and CD4^+^ IL-17^+^ T cells (*p* < 0.05, d5 p.i.) in contrast to IL-4^+^, IL-10^+^ and Foxp3^+^ CD25^+^ CD4^+^ T cells (Additional file 2: Figure S2). These experiments suggested that PαS MSC treatment modulated adaptive immunity by impairing pro-inflammatory IL-17 and IFN-γ expressing T cell subsets.

### PαS MSC improve pneumonia survival and do not increase bacterial load

**Fig. 7:**
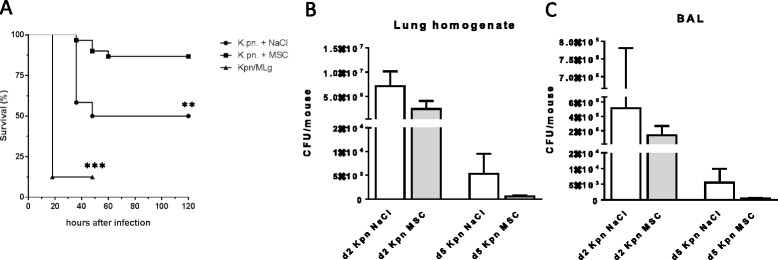
PαS MSC improve survival and do not impair bacterial clearance after *K. pneumoniae* infection. Survival of *K. pneumoniae*-infected animals treated with NaCl, PαS MSC or MLg fibroblasts (**a**). Bacterial load (colony forming units, CFU) of lung homogenate (**b**) and BAL (**c**) of *K. pneumoniae*-infected (Kpn) animals treated with PαS MSC or NaCl at 48 h and d5 p.i. (**a**) *n* ≥ 8, (**b**, **c**) mean ± SEM; *n* ≥ 3 per group. **p < 0.01, ***p < 0.001 versus mock-treated infected controls. Data from *n* ≥ 2 (A-C) experiments

PαS MSC treatment after respiratory *K. pneumoniae* infection resulted in significantly improved overall survival in comparison to mock-treated (NaCl only) animals (Fig. 7a). To assess whether the positive results of PαS MSC may be related to an unspecific cellular effect we performed experiments with MLg fibroblasts instead of PαS MSC. Most MLg fibroblast-treated animals died within 24 h p.i. resulting in significantly reduced survival in comparison to both PαS MSC and NaCl-only treated animals (*p* < 0.01; Fig. 7a).

With respect to the markedly impaired respiratory inflammation and reduced numbers of pro-inflammatory T cells PαS MSC may exert immunosuppressive activity and might increase bacterial loads after infection. Accordingly, we determined the bacterial loads in lung homogenates and BAL at different time points after *K. pneumoniae* infection (Fig. 7b-c). The results consistently showed that PαS MSC treatment did not result in increased bacterial loads in lung homogenates or in BAL, indicating that PαS MSC are unlikely to impair antibacterial immunity.

## Discussion

In this study we report on the immunomodulatory capacity of prospectively defined MSC in a preclinical mouse model of acute bacterial pneumonia. Many studies have highlighted the immunomodulatory capacity of MSC but most, if not all studies have used heterogeneous mixtures of starting cells for the in vitro expansion of MSC. Recently Matsuzaki et al. has identified a BM-derived MSC subset, expressing PDGFRα and Sca-1, so-called PαS cells, that are highly enriched for CFU-Fs with differentiation potential [7]. Here we have demonstrated that these prospectively defined PαS cells exhibited marked immunomodulatory capacity in vivo. The prospective identification of MSC based on surface markers facilitates the development of standardized protocols and additionally improves the comparability of scientific studies [4, 8]. In accordance with Morikawa et al., we have found that cultured PαS cells uniformly expressed conventional MSC markers CD29, CD49e and SCA-1 and were positive for CD90 and CD105 [7].

Our results using prospectively defined MSC showed that expanded PαS cells effectively suppressed acute lung injury and acute alveolitis caused by respiratory K. pneumoniae. We selected a *Klebsiella* species as the pathogen because they represent an important group of bacteria that causes life threatening nosocomial infections [28]. Furthermore, multidrug resistant *Klebsiella* strains are of increasing clinical relevance worldwide due to limited treatment options [29]. We showed that PαS MSC treatment significantly inhibited alveolar protein leakage, granulocytosis and TNF-α production indicating a marked in vivo anti-inflammatory capacity. Furthermore, PαS MSC treatment inhibited not only acute lung injury but additionally suppressed expansion of pro-inflammatory T helper subsets expressing IL-17 and IFN-γ in the post-acute pneumonia phase. Our flow cytometry analysis of respiratory DC subsets provides a rationale for understanding the marked inhibition of IL-17 and IFN-γ expressing T helper cells. Our results suggested that PαS MSC treatment effectively inhibited accumulation and CD86 upregulation on respiratory CD103^+^ DC. Different reports have highlighted the critical role of CD103^+^ DC for the expansion of IL-17 and IFN-γ driven T effector cell responses [30, 31].

Various studies have investigated the effect of MSC in models of LPS- or bleomycin-induced lung injury and have reported significant amelioration after lung injury [18, 32, 33] as well as a significant survival benefit in MSC-treated animals [18, 33].

Only a few studies have investigated the therapeutic activity of MSC in pneumonia models with live bacteria [19, 20, 34]. Gupta et al. and Kim et al. reported that intratracheal MSC treatment improved survival and bacterial clearance in murine *Escherichia coli* pneumonia [19, 20]. Here we have extended these findings by using prospectively defined PαS MSC in a *K.* pneumonia-infected animal model. Independent animal models employing live bacteria are of clinical relevance because there may be concerns that “immunosuppressive” MSC might promote bacterial growth due to an impairment of host defences. Our results using prospectively defined MSC are in agreement with other data indicating that intratracheal MSC therapy during infectious pneumonia promotes rather than inhibits bacterial clearance [19]. Matthay et al. has suggested that the antibacterial effect of MSC are due to the upregulation of the antibacterial proteins lipocalin 2 and LL37 [19, 35]. Recent evidence by Devaney et al. further supports this finding in a rat E. coli pneumonia model. In their study, MSC-treatment significantly reduced acute lung injury, improved overall survival and decreased lung bacterial load. With respect to the potential anti-bacterial activity of MSC they reported enhanced macrophage phagocytic capacity and increased lung and systemic concentrations of the antimicrobial peptide LL37 in MSC-treated animals [34]. In agreement with these findings, our histopathological analyses indicated a strikingly reduced number of bacteria in the lungs of MSC-treated animals. However, mean values of bacterial loads were decreased in MSC-treated animals at d2 and d5 p.i. but these differences were statistically not significant due to the inter-individual variation. One may speculate that the antibacterial activity of MSC may play a key role in models with live bacteria. With respect to the immunomodulatory activity of MSC, several studies using different models have reported that MSC induced increased levels of IL-10 or Foxp3+ regulatory T cells [18, 36, 37]. However, in our study both IL-10 production as well as Foxp3+ CD4+ regulatory T cell frequencies were unaffected after MSC-therapy. Recently, Bustos et al. reported that pre-activation of human MSC with serum from patients with ARDS markedly increased their therapeutic capacity in a murine pneumonia model concomitant with increased IL-10 and IL-1 receptor antagonist levels [38]. Therefore, different levels of MSC pre-activation in different animal models may impact on their immunomodulatory capacity. With respect to the immunosuppressive capacity of MSC we observed significantly reduced CD86 surface expression on lung CD103+ DC in MSC-treated animals early after infection. CD86 represents a major T cell costimulatory molecule that is critically involved in T cell activation [39]. However, CD86 suppression was not significantly suppressed on CD11b + DC which may be related to different costimulatory molecule expression kinetics or differential migratory kinetics of CD11b + DC into inflamed tissue areas.

In summary, we have investigated the in vivo immunomodulatory capacity of prospectively defined, purified PαS MSC in a clinically relevant *K. pneumoniae* model. PαS MSC efficiently suppressed acute lung injury and promoted overall pneumonia survival. Flow cytometry analysis revealed impaired lung DC infiltration and CD103^+^ DC maturation representing known key drivers of T cell-mediated inflammation, which provided a rationale for the sustained anti-inflammatory effects of MSC therapy. These findings support the potential of using prospectively defined MSC-based therapies for patients with acute bacterial pneumonia and provide new insight into the immunomodulatory capacity of PαS cells.

## Conclusions

This experimental animal study has analyzed the in vivo immunomodulatory activity of prospectively defined MSC in acute bacterial pneumonia. In most published studies, MSC have been retrospectively defined based on their plastic adherent properties leading to heterogeneous cell populations. Our results indicate that intratracheal application of MSC effectively inhibit K.pneumoniae-induced acute lung injury and respiratory inflammation and provide additional evidence for the therapeutic activity of MSC in bacterial pneumonia.

## Additional files

Additional file 1: Figure S1. Gating strategy for the identification of respiratory leukocyte subsets. Respiratory leukocytes were identified using CD45 surface expression. Respiratory lymphocytes were identified according to low side scatter characteristics and subsequently discriminated into CD19+ B cells, NK1.1+ NK cells, CD3+ γδ TCR+ gamma-delta T cells, CD3+ CD4+ T cells and CD3+ CD8+ T cells. Granulocytes and alveolar macrophages were subsequently identified out of CD45+ leukocytes based on CD11b and GR-1 expression (granulocytes) and CD11c and Siglec F surface expression (alveolar macrophages). DC subsets were subsequently identified out of CD45+ leukocytes based on CD11c expression in SiglecF/NK1.1/Gr1 negative cells to exclude contamination by alveolar macrophages, NK cells and granulocytes. This CD11c positive fraction was further discriminated into CD103+ DC and CD11b DC based on MHC-class II, CD103 and CD11b expression as well as in pDC based on 120 g8 expression and absence of CD11b. Representative figure from n > 10 experiments. (PDF 382 kb) Additional file 2: Figure S2. Modulation of respiratory CD4+ CD25+ Foxp3+ regulatory T cells after PαS MSC treatment. Representative flow cytometry analysis of CD4+ CD25+ Foxp3+ regulatory T cells in K. pneumoniae-infected animals treated with PαS MSC versus mock-treated infected animals (A, d5 p.i.). Iso refers to isotype-matched fluorescence minus-one controls to assess background staining. The frequency in % refers to the frequency of CD4+ CD25+ Foxp3+ T cells among all CD4+ T cells. Relative frequencies and absolute numbers of respiratory CD4+ CD25+ Foxp3+ regulatory T cells in K. pneumoniae-infected (Kpn) animals treated with PαS MSC versus mock-treated infected animals (**B**). Mean ± SEM; n ≥ 4. Data from *n* = 2 (**A**-**B**) experiments. (PDF 238 kb)

## Abbreviations

ATCC: American type culture collection
BM: Bone marrow
BAL: Bronchoalveolar lavages
CFU-F: Colony-forming unit-fibroblasts
DC: Dendritic cells
HSC: Hematopoietic stem cells
K. pneumoniae: Klebsiella pneumoniae
MSC: Mesenchymal stem cells
MLg: Mouse lung fibroblasts
PαS: PDFGRa + SCA1+ CD45- TER119-
