# Supplementary figures and images for: Prospectively defined murine mesenchymal stem cells inhibit Klebsiella pneumoniae-induced acute lung injury and improve pneumonia survival
Source: Respir Res. 2015 Oct 6;16:123. doi: 10.1186/s12931-015-0288-1 (PMC4594670; doi:10.1186/s12931-015-0288-1)

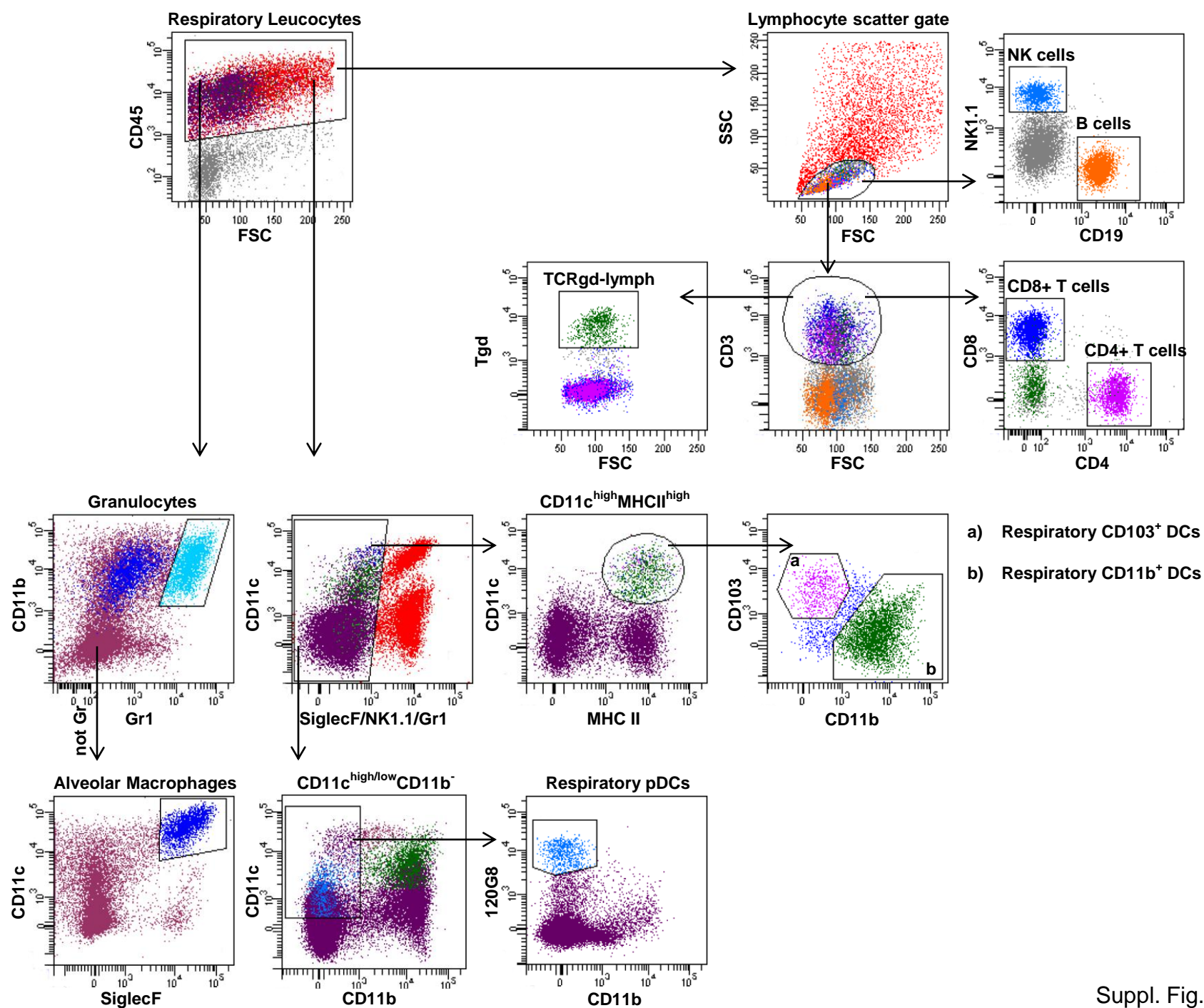

Suppl. Fig. 1

Supplement: Additional file 1: Figure S1. — Gating strategy for the identification of respiratory leukocyte subsets. Respiratory leukocytes were identified using CD45 surface expression. Respiratory lymphocytes were identified according to low side scatter characteristics and subsequently discriminated into CD19+ B cells, NK1.1+ NK cells, CD3+ γδ TCR+ gamma-delta T cells, CD3+ CD4+ T cells and CD3+ CD8+ T cells. Granulocytes and alveolar macrophages were subsequently identified out of CD45+ leukocytes based on CD11b and GR-1 expression (granulocytes) and CD11c and Siglec F surface expression (alveolar macrophages). DC subsets were subsequently identified out of CD45+ leukocytes based on CD11c expression in SiglecF/NK1.1/Gr1 negative cells to exclude contamination by alveolar macrophages, NK cells and granulocytes. This CD11c positive fraction was further discriminated into CD103+ DC and CD11b DC based on MHC-class II, CD103 and CD11b expression as well as in pDC based on 120 g8 expression and absence of CD11b. Representative figure from n > 10 experiments. (PDF 382 kb) [file 12931_2015_288_MOESM1_ESM.pdf]

A

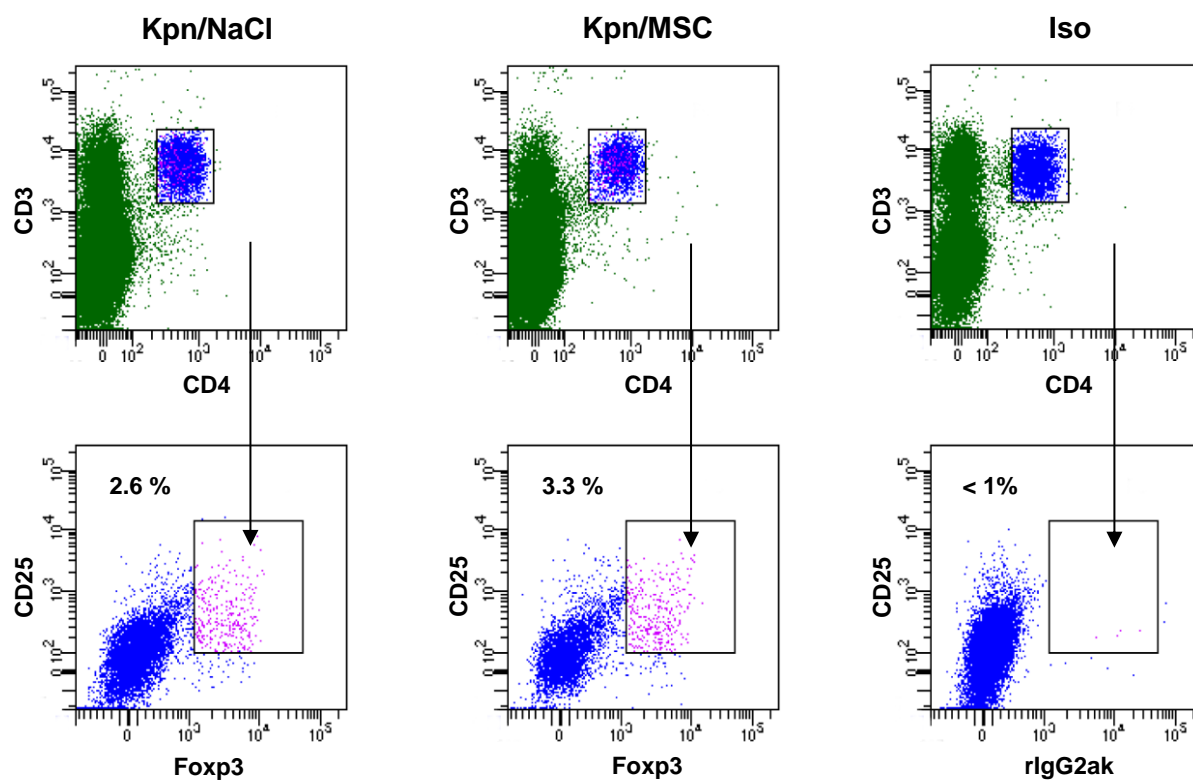

B

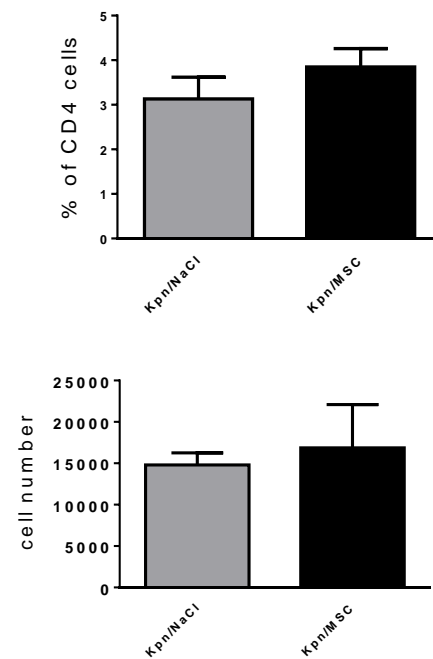

Supplement: Additional file 2: Figure S2. — Modulation of respiratory CD4+ CD25+ Foxp3+ regulatory T cells after PαS MSC treatment. Representative flow cytometry analysis of CD4+ CD25+ Foxp3+ regulatory T cells in K. pneumoniae-infected animals treated with PαS MSC versus mock-treated infected animals (A, d5 p.i.). Iso refers to isotype-matched fluorescence minus-one controls to assess background staining. The frequency in % refers to the frequency of CD4+ CD25+ Foxp3+ T cells among all CD4+ T cells. Relative frequencies and absolute numbers of respiratory CD4+ CD25+ Foxp3+ regulatory T cells in K. pneumoniae-infected (Kpn) animals treated with PαS MSC versus mock-treated infected animals (B). Mean ± SEM; n ≥ 4. Data from n = 2 (A-B) experiments. (PDF 238 kb) [file 12931_2015_288_MOESM2_ESM.pdf]
